# Opportunities Revealed for Antimicrobial Stewardship and Clinical Practice with Implementation of a Rapid Respiratory Multiplex Assay

**DOI:** 10.1128/JCM.00861-19

**Published:** 2019-09-24

**Authors:** Zoe F. Weiss, Cheston B. Cunha, Alison B. Chambers, Audrey V. Carr, Cleo Rochat, Mariska Raglow-Defranco, Diane M. Parente, Aimee Angus, Leonard A. Mermel, Latha Sivaprasad, Kimberle Chapin

**Affiliations:** a Rhode Island Hospital, Department of Medicine, Warren Alpert Medical School of Brown University, Providence, Rhode Island, USA; b Rhode Island Hospital, Division of Infectious Diseases, Warren Alpert Medical School of Brown University, Providence, Rhode Island, USA; c Lifespan Biostatistics Core, Rhode Island Hospital, Providence, Rhode Island, USA; d Warren Alpert Medical School of Brown University, Providence, Rhode Island, USA; e Department of Pharmacy, Miriam Hospital, Providence, Rhode Island, USA; f Division of Pathology and Laboratory Medicine, Rhode Island Hospital, Providence, Rhode Island, USA; g Rhode Island Hospital, Executive Administration, Providence, Rhode Island, USA; h Division of Pathology and Laboratory Medicine, Rhode Island Hospital, Providence, Rhode Island, USA; UNC School of Medicine

**Keywords:** antibiotic stewardship, influenza, multiplex PCR, respiratory pathogens

## Abstract

Few studies assess the utility of rapid multiplex molecular respiratory panels in adult patients. Previous multiplex PCR assays took hours to days from order time to result. We analyze the clinical impact of switching to a molecular assay with a 3-h test-turnaround-time (TAT). We performed a retrospective review of adult patients who presented to our emergency departments with respiratory symptoms and had a respiratory viral panel (xTAG RVP; RVP) or respiratory pathogen panel (ePlex RP; RPP) within 48 h of presentation. The average TATs for the RVP and RPP were 27.9 and 3.0 h, respectively (*P* < 0.0001). In RVP-positive and RPP-positive patients, 68.9 and 44.5% of those with normal chest imaging received antibiotics (*P* = 0.013), while 95.4 and 89.6% of those with abnormal imaging received antibiotics, respectively (*P* = 0.187). There was no difference in antibiotic duration in RVP-positive and RPP-positive patients with abnormal chest imaging (6.2 and 6.0 days, respectively; *P* = 0.923) and normal chest imaging (4.5 and 4.3 days, respectively; *P* = 0.922). Fewer patients were admitted in the RPP-positive compared to the RVP-positive group (76.9 and 88.6%, respectively; *P* = 0.013), while the proportion of admissions were similar among RPP-negative and RVP-negative patients (85.3 and 87.1%, *P* = 0.726). Switching to a multiplex respiratory panel with a clinically actionable TAT is associated with reduced hospital admissions and, in admitted adults without focal radiographic findings, reduced antibiotic initiation. Opportunities to further mitigate inappropriate antibiotic use may be realized by combining rapid multiplex PCR with provider education, clinical decision-care algorithms, and active antibiotic stewardship.

## Abstract (precis)

Few studies assess the utility of rapid multiplex molecular respiratory panels in adult patients. Previous multiplex PCR assays took hours to days from order time to result. We analyze the clinical impact of switching to a molecular assay with a 3-h test-turnaround-time (TAT).

## INTRODUCTION

Respiratory multiplex PCR panels are highly sensitive and specific, allowing clinicians to identify likely causal organisms in patients with symptoms suggestive of respiratory infection (1, 2). Identifying a viral pathogen should ideally reduce unwarranted antibiotic use, and thus subsequently the burden of colonization or infection from multidrug-resistant organisms and Clostridium difficile (1).

The impact of multiplex respiratory panels used in hospitalized adults has been mixed, with either minimal (3, 4) or no improvement in reducing antibiotic use (5–11), even among those without infiltrates on chest radiographs (12–14) or in conjunction with procalcitonin (15). Little has been published on the clinical impact of rapid multiplex PCR (<4 h) in hospitalized adult patients. In one study of both adult and pediatric patients, reduced antibiotic prescribing was observed; however, study personnel verbally reported test results and questioned providers about their decision to withhold or prescribe antibiotics (16). Another study comparing rapid (average, 2.3 h) to less-timely multiplex PCR (average, 37 h) in adults found more patients treated with single doses or a brief course of antibiotics (17). Other adult studies have shown reduced antibiotic use, but small sample size (18), unique site-specific algorithms (16, 18–21), turnaround times (TATs) of 9 to 30 h (8, 12, 22, 23), or analysis of just the influenza component (24) make these findings difficult to generalize to multiplex results.

Lack of improvement in antibiotic utilization despite viral confirmation in the inpatient setting may, in part, be due to prolonged TAT (17, 25). Multiplex PCR panels that previously took hours to days to obtain results (13, 22, 23) were performed in batches or only after initial influenza testing (24) and often provided results to providers after antibiotics were already initiated. Our institution previously used the Respiratory Viral Panel (xTAG RVP [RVP]), with results available between 12 h and 3 days depending on the collection time and daily batch testing (26). The Respiratory Pathogen Panel (ePlex RP [RPP]) was subsequently instituted (27) and performed as specimens were received during all shifts. We hypothesized that switching from the RVP to the RPP would result in reduced antibiotic initiation and duration in admitted patients, particularly in patients with positive viral testing and chest imaging without infiltrates.

## MATERIALS AND METHODS

### Study setting.

This was a retrospective study conducted at two acute large tertiary care teaching hospitals within the same academic and health care system in Rhode Island. Rhode Island Hospital is a 713-bed tertiary care center, and The Miriam Hospital is a 247-bed community hospital. The study protocol was approved by the institutional review board for both institutions.

### RVP and RPP tests.

The previously instituted RVP (Luminex Corporation, Austin, TX) detected influenza A virus (H1, H3, and H5), influenza B virus, respiratory syncytial virus (RSV) A, RSV B, coronavirus (NL63, OC43, HKU1, and 229E), parainfluenza virus (types 1 to 4), metapneumovirus, rhinovirus, enterovirus, and adenovirus (28). The sensitivity and specificity were 91.2 and 99.7%, respectively (29), with reported TATs between 12 and 24 h (30). Of note, this test is no longer commercially available in the United States and has been replaced by the reduced step version, namely, the Luminex NxTAG respiratory panel. This assay is still, however, a multistep batch mode assay, corresponding to prolonged TATs depending on the timing of sample collection.

The recently implemented RPP (GenMark Diagnostics, Inc., San Diego, CA) includes adenovirus, coronavirus (229E, HKU1, NL63, and OC43), influenza A virus (H1, 2009 H1N1, and H3), influenza B virus, RSV (A and B), parainfluenza virus (types 1 to 4), human metapneumovirus, and enterovirus/rhinovirus, as well as three atypical pathogens, Bordetella pertussis, Mycoplasma pneumoniae, and Chlamydophila pneumoniae (31). The RPP has 100% concordance with laboratory developed testing (31), >95% agreement with an alternative multisyndromic respiratory panel, and a reported TAT of 2 to 4 h (32).

At our institutions, multiplex viral testing is recommended for inpatients or those likely to be admitted, whereas rapid influenza A/B PCR testing (Cepheid, Sunnyvale, CA) is recommended for outpatients or those likely to be discharged from the emergency department (ED). A strict testing algorithm is not enforced. Respiratory panels are performed at both institutions in the microbiology lab. The results are immediately released upon assay completion in the electronic medical record.

### Patient selection.

Patients included in our analysis were ≥18 years old, assessed in one of the two EDs, and admitted to one of the two hospitals during November 2016 and February 2017 (RVP group) or November 2018 and February 2019 (RPP group) with ICD-10 coded diagnoses indicating lower respiratory symptoms with a nasopharyngeal-swab specimen submitted for RVP or RPP within 48 h of presentation. Two months from consecutive influenza seasons were chosen due to the high volume of samples, respectively. Patients were excluded if they were on outpatient antibiotics prior to admission, received antibiotics for other indications (e.g., concurrent cellulitis, urinary tract infection, bacteremia with positive blood cultures, ventilator-associated pneumonia, neutropenic fever, or aspiration pneumonia with an abnormal swallow evaluation) or were comfort-care only.

### Primary and secondary outcomes.

Primary outcomes were antibiotic initiation and duration, stratified by normal or abnormal chest imaging. Secondary outcomes included inpatient admissions, length of stay (LOS), death during hospitalization, allergic reactions, the development of Clostridium difficile infection within 30 days, and readmission within 30 days.

### Data collection.

Data extracted from electronic medical records included the choice, duration, and administration time of antibiotics. Length of therapy (LOT) was calculated from the date of the first to the last administered dose. For patients discharged on antibiotics, the date of the last dose was extrapolated from the days of therapy prescribed at discharge. All initial chest imaging (including chest x-rays and chest computed tomography scans) were reviewed and categorized as “normal” or “abnormal” based on the dictated radiology interpretation. Imaging was labeled normal if the interpretation indicated no acute pulmonary process (e.g., clear lung fields, no acute cardiopulmonary process, or chronic emphysematous changes without superimposed airspace disease). Radiology interpretations indicating an acute pulmonary process were considered “abnormal.” To verify our laboratory’s TATs, the time of specimen collection to the time of result availability in the electronic medical record (EMR) was recorded for each sample.

### Statistical analysis.

Clinical and laboratory data for all patients who met inclusion criteria were used in all statistical analysis and for all hypothesis testing. All statistical tests were run using Proc Glimmix, allowing for fitting of both general and generalized linear models (33, 34), as well as deriving *P* values for hypothesis tests or estimated mean comparisons (SAS v9.2; SAS Institute, Inc., Cary, NC). For primary and secondary outcomes, a generalized linear model for lognormal, normal, or binary outcomes was used to analyze demographics and confounding variables to test for differences between respiratory test periods (RPP versus RVP).

Antibiotic use was assessed using three different metrics: whether or not antibiotics were prescribed, whether antibiotics were given before test resulted, and the number of days prescribed. A generalized linear model for binary outcomes was used to analyze and estimate the proportion of patients who received antibiotics by respiratory testing (RPP and RVP) and chest imaging results (normal/abnormal). An interaction term was included in the model to allow for differences by level of chest imaging result (normal/abnormal). This analysis was repeated to test the proportion of patients who received antibiotics before EMR test result availability.

A negative binomial distribution was used to model days of antibiotics by viral test and chest imaging results. An interaction term was also included to allow for differences in the relationship between the viral test used and antibiotic days, with the chest imaging result. A subanalysis was performed to test whether antibiotics were subsequently discontinued; analysis was run on the subgroup of patients who received antibiotics before EMR test result availability.

The proportions of patients who tested positive, who were admitted, who had viral testing performed in the ED, who underwent 30-day readmittance, who experienced in-hospital mortality, who were admitted to the intensive care unit (ICU), who were influenza-positive and treated with antivirals, and for whom C. difficile studies were positive were analyzed to test for indirect systemic differences between study periods (RPP versus RVP). We also analyzed the length of stay based on the respiratory test used with an interaction term for antibiotics administered.

Classic sandwich estimation was used to adjust for any model misspecification. A family-wise alpha was maintained at 0.05 using the Holm adjustment for multiple comparisons. Adjusted *P* values are reported unless otherwise stated. All statistical models were run using Proc Glimmix, allowing for modeling of both general and generalized linear models, as well as deriving *P* values for model fixed effects or estimated mean comparisons (SAS v9.2).

## RESULTS

**FIG 1:**
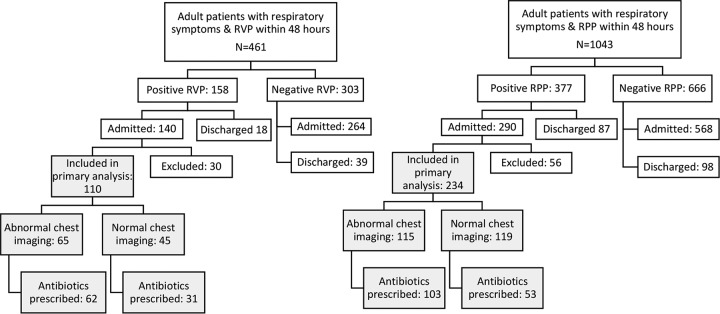
Flow chart of sample inclusion criteria. Patients used primarily for analysis are highlighted in gray.

In all, 461 and 1,043 patients who had RVP and RPP testing, respectively, were identified and screened for study inclusion. Of these, 110 (26.6%; 95% confidence interval [CI], 22.6 to 31.1) and 234 (26.0%; 95% CI, 23.2 to 29; *P* = 0.808) were admitted patients tested positive and were included in the RVP and RPP groups, respectively, for the primary analysis (Fig. 1). The mean age in the RVP group was 70.5 years (95% CI, 67.1 to 74.0 years; age distribution, 18 to 40 years; *n* = 10 [9.2%]; 41 to 60 years, *n* = 16 [14.6%], and >60 years, *n* = 84 [76%]) and in the RPP group was 70.2 years (95% CI, 68.1 to 72.3; age distribution, 18 to 40 years, *n* = 14 [6.0%]; 41 to 60 years, *n* = 39 [16.7%], and >60 years, *n* = 181 [77.4%]). The average TATs for positive RVP and RPP results were 27.9 h (95% CI, 24.4 to 31.9 h) and 3.0 h (95% CI, 2.9 to 3.2 h), respectively (*P* < 0.0001). Patients who tested positive using the RVP were more likely to be in an ICU compared to the RPP-positive group (27.3 and 17.5%, respectively; *P* = 0.039), and a smaller proportion of patients with positive RVP testing had asthma (12.7 and 23.1%, respectively; *P* = 0. 027) (Table 1). Influenza A virus and entero/rhinovirus were the most commonly detected pathogens in the RVP- and RPP-positive groups (Table 2).

**TABLE 1:** Baseline patient characteristics^*a*^

Patient characteristic	RVP		RPP		*P*
	No. of subjects^*b*^ (*n* = 110)	% (95% CI)	No. of subjects (*n* = 234)	% (95% CI)	
Mean age (yr)	70.5	(67.1–74.0)	70.2	(68.1–72.3)	0.862
Female	64	58.2 (48.8–67.1)	124	53.0 (46.6–59.3)	0.368
COPD	50	45.5 (36.4–54.8)	104	44.4 (38.2–50.9)	0.861
Asthma	14	12.7 (7.7–20.4)	54	23.1 (18.1–28.9)	0.027
Immunosuppressed	13	11.8 (7.0–19.3)	36	15.4 (11.3–20.6)	0.379
Transplant	2	1.8 (0.5–7.0)	5	2.1 (0.9–5.0)	0.846
ICU level of care	30	27.3 (19.8–36.4)	41	17.5 (13.2–23)	0.039

a *P* values were calculated from the generalized linear models for normal distribution (age) and binary variables. *n*, total number of subjects.

b Except as noted for the mean age in column 1.

**TABLE 2:** Pathogens detected

Pathogen^*a*^	No. (%) of subjects	
	RVP	RPP
Adenovirus	0 (0)	6 (2.6)
Coronavirus	19 (17.3)	18 (7.7)
Influenza A virus	26 (23.6)	81 (34.6)
Influenza B virus	0 (0)	13 (5.6)
Human metapneumovirus	7 (6.4)	16 (6.8)
Entero/rhinovirus	35 (31.8)	61 (26.0)
Parainfluenza virus	16 (14.6)	11 (4.7)
RSV	14 (12.7)	37 (15.8)
Chlamydophila pneumoniae	NA	1 (0.4)
Mycoplasma pneumoniae*	NA	3 (1.3)
Bordetella pertussis*	NA	0 (0)
One pathogen	103 (93.6)	223 (95.3)
Two pathogens	7 (6.4)	10 (4.7)

a *, Not included in the Luminex xTAG respiratory viral panel (RVP) assay. NA, not applicable.

### Antibiotic usage.

**FIG 2:**
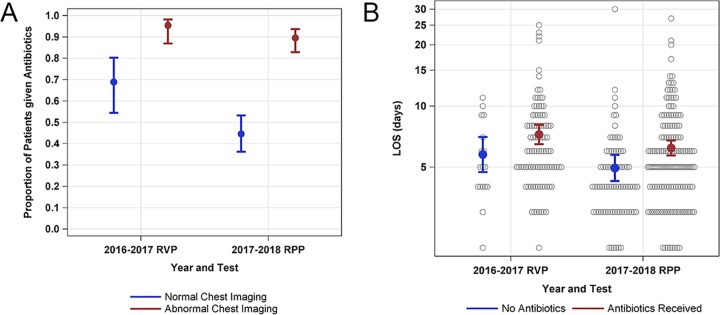
(A) Model estimates for proportions of admitted patients initiated on antibiotics based on positive RVP or RPP results and findings on chest imaging (normal chest imaging or abnormal chest imaging). Bars represent 95% confidence intervals. (B) Model estimates for the length of stay (LOS) in days in patients admitted patients with positive RVP or RPP testing based on whether or not antibiotics were given. Bars represent the 95% CI values. Gray circles represent individual patients’ LOS.

In the RVP and RPP groups, antibiotic initiation estimated from the model was lower in patients with normal chest imaging compared to abnormal chest imaging (57.2% [48.1 to 5.8] and 93.0% [87.4 to 96.2], respectively; *P* < 0.001). In patients with normal imaging, antibiotic initiation was significantly lower in the RPP-positive group compared to the RVP-positive group (44.5% [95% CI, 35.8 to 53.6] and 68.9% [95% CI, 54.0 to 80.7], respectively; *P* = 0.013; Fig. 2A). In patients with abnormal chest imaging, there were no differences in antibiotic initiation between those with positive RVP and RPP test results (95.4% [95% CI, 86.6 to 98.5] and 89.6% [95% CI, 82.5 to 94.0], respectively; *P* = 0.187).

For patients with normal imaging, the proportion that received antibiotics before their test resulted in the EMR was lower in the RPP-positive group than in the RVP-positive group (54.7% [95% CI, 38.8 to 69.7] and 96.8% [95% CI, 79.7 to 99.6], respectively; *P* = 0.007). For patients with abnormal chest imaging, the proportion that received antibiotics before their test resulted was lower in the RPP-positive group than the RVP-positive group (81.6% [95% CI, 73.9 to 87.4] and 100%, respectively; *P* < 0.001).

There was no difference in antibiotic days between the RVP-positive and RPP-positive patients with abnormal chest imaging who received antibiotics before the results of the respiratory test was available (6.2 days [95% CI, 5.3 to 7.3] and 6 days [95% CI, 5.4 to 6.6], respectively; *P* = 0.923) and with normal chest imaging (4.5 days [95% CI, 3.5 to 5.7] and 4.3 days [95% CI, 3.4 to 5.4], respectively; *P* = 0.922).

In patients who received antibiotics, there was no difference in atypical coverage in the RVP-positive and RPP-positive groups (81.7% [72.5 to 88.3] and 80.1% [73.1 to 85.7], respectively; *P* = 0.76; Table 3). Four patients positive for atypical bacteria (three with Mycoplasma pneumoniae and one with Chlamydophila pneumoniae) with the RPP received appropriate coverage.

**TABLE 3:** Number and proportion of patients who were exposed to each antibiotic, followed by the weighted proportion of antibiotic days^*a*^

Antibiotic treatment	RVP (*n* = 93)			RPP (*n* = 156)		
	No. of subjects	Exposed (%)	Weighted (%)	No. of subjects	Exposed (%)	Weighted (%)
Penicillins						
Piperacillin-tazobactam	34	36.6	18.0	40	25.6	15.0
Ampicillin-sulbactam*	7	7.5	2.9	12	7.7	5.1
						
Cephalosporins						
Ceftriaxone	41	44.1	14.6	47	30.1	13.8
Cefepime	8	8.6	2.6	7	4.5	1.5
Vancomycin	39	41.9	15.7	51	32.7	16.6
Atypical coverage**	76	81.7	42.4	125	80.1	45.9
Azithromycin	70	75.3	33.6	91	58.3	28.2
Doxycycline	5	5.4	2.2	13	8.3	4.9
Fluoroquinolones	18	19.4	6.6	41	26.3	12.8
Other	8	8.6	3.8	8	5.1	2.1

a The weighted proportion was calculated by dividing the total patient days of each respective antibiotic by cumulative antibiotic days for each time period. *, Patients who received amoxicillin-clavulanate were also included in this category. **, Atypical coverage includes azithromycin, doxycycline, or fluoroquinolones.

### Secondary outcomes.

In patients with RVP- or RPP-negative results, there were no differences in the proportion hospitalized (85.3% [95% CI, 82.4 to 87.8] and 87.1% [95% CI, 82.9 to 90.5], respectively; *P* = 0.726). However, those who had positive testing were less frequently admitted from the ED in the RPP group compared to the RVP group (76.9% [95% CI, 72.4 to 80.9] and 88.6% [95% CI, 82.6 to 92.7], respectively; *P* = 0.013).

For patients who did not receive antibiotics, there were no differences in length of stay (LOS) between RVP-positive and RPP-positive patients (5.8 days [95% CI, 4.7 to 7.1 days] and 4.9 days [95% CI, 4.2 to 5.8 days], respectively; *P* = 0.483). For patients initiated on antibiotics, a decrease was seen in LOS between RVP-positive and RPP-positive groups (7.2 days [95% CI, 6.4 to 8.1 days] and 6.2 days [95% CI, 5.7 to 6.8 days], respectively; unadjusted *P* = 0.048 and adjusted [for abnormal imaging] *P* = 0.195; Fig. 2B). Of patients who had influenza A or B, 88.5% in the RVP group and 90.3% in the RPP group received oseltamivir (*P* = 0.78). No allergic reactions to antibiotics were observed in either group, and there were no significant differences in positive C. difficile testing, 30-day hospital readmission, or death during hospitalization (Table 4).

**TABLE 4:** Secondary outcomes^*a*^

Outcome	RVP count	%RVP (95% CI)	RPP count	%RPP (95% CI)	*P*
Admission with a positive test	140/158	88.6 (82.6–92.7)	290/377	76.9 (72.4–80.9)	0.013
Admission with a negative test	264/303	87.1 (82.9–90.5)	568/666	85.3 (82.4–87.8)	0.726
Test performed in the ED	49/110	44.6 (35.5–54.0)	191/234	81.6 (76.1–86.1)	<0.001
30-day readmittance	12/110	10.9 (6.3–18.3)	35/234	15.0 (10.9–20.1)	0.311
In-hospital death	4/110	3.6 (1.4–9.3)	4/234	1.7 (0.6–4.5)	0.281
C. difficile infection	0/110	NA^*b*^	3/234	1.3 (0.4,3.9)	NA
Allergic reactions	0/110	NA	0/234	NA	NA
Appropriate oseltamivir^*c*^	23/26	88.5 (69.5–96.3)	84/93	90.3 (82.3–95.0)	0.781

a Counts are expressed as the number of affected subjects/total number of subjects examined.

b NA, not applicable.

c Appropriate oseltamivir is defined as the use of oseltamivir in patients testing positive for influenza A or B.

## DISCUSSION

Implementation of respiratory pathogen testing with results available in 3 h was associated with a reduction in both antibiotic initiation and hospital admissions, with no observable change in LOT or LOS. To date, most clinical impact studies of respiratory multiplex PCR assays with positive outcomes have been in pediatric patients (35–37) or have focused on influenza testing and oseltamivir use only (38, 39). The impact of syndromic testing on antibiotic use in adults has been more variable. In one meta-analysis (40), an overall reduction in LOS was observed, with no differences in hospital admissions. No significant reduction in antibiotic initiation or LOT were observed (40). However, only four of the studies referenced addressed antibiotic use with assays that had a TAT of <4 h. Three found a reduction in antibiotic LOT (17, 19, 20), with one showing a decrease in antibiotic initiation in the subgroup analysis of patients whose tests resulted prior to antibiotic administration (17). Thus, assay TAT for respiratory pathogens alone has not been clearly established as a factor in reducing antibiotic use. This is important, since several multiplex syndromic assays exist, but the ability to perform the test in random access fashion as the specimen comes to the lab, rather than by daily batch testing, with reliable TAT, as well as bacterial targets, may be critical to moving the needle on antibiotic use.

A consistent rapid result from a multiplex assay may allow for coordinated decision making by the provider with other available clinical data, such as chest radiographs or biomarker data. Our study builds on previous findings that chest imaging is a strong predictor of antibiotic administration, even though radiography does not reliably distinguish between bacterial and viral infections (13). We demonstrated a clear reduction in antibiotic initiation in patients who had a rapid RPP and normal chest imaging. This may reflect an increasing dependence on confirmatory diagnostic testing to enhance provider confidence in withholding antibiotics. In patients with abnormal imaging, antibiotic initiation was similar between the RVP-positive and RPP-positive groups, and in RPP-positive patients with abnormal chest imaging, 81.6% of those prescribed antibiotics were initiated before the test resulted. In all patients, once empirical antibiotics were initiated, some within minutes of the test result, neither a reduction in duration nor early discontinuation was observed.

Physicians may not feel comfortable stopping antibiotics that were initiated based on the clinical assessment they were not present for in the ED (22) or because patients with viral infections are clinically “improving” while on antibiotics. As such, it may be reasonable for ED physicians to withhold empirical antibiotics in nonseptic patients, without evidence of focal infiltrate, until the results of the multiplex assay become available.

Multiplex PCR may also help guide the choice of empirical therapy. Azithromycin was the most commonly prescribed antibiotic in both time periods with atypical coverage provided to 80% of patients in the RVP-positive and RPP-positive groups. However, fewer than 2% of patients had an atypical organism identified by RPP, raising the question of whether clinicians were aware that these pathogens were available on the RPP assay. Physicians may consider withholding atypical antibiotic coverage except in cases where Legionella pneumophila is clinically suspected.

In the setting of abnormal chest imaging, the use of multiplex PCR alone for guiding antibiotic therapy is less immediately clear (41), since a confirmed viral pathogen may be insufficient evidence for providers to withhold or stop antibiotics, given concerns regarding potential bacterial coinfection (8, 12). Though not currently performed at our institution, the implementation of biomarkers, such as procalcitonin, as part of a clearly defined diagnostic algorithm, may help determine whether bacterial coinfection is likely, and earlier discontinuation possible, though its use is controversial (15, 21, 42). Decision support tools within the EMR, clinician education, diagnostic management teams to aid in the development of test algorithms and interpretations, and real-time stewardship may improve antibiotic utilization and diagnostic stewardship efforts (43–45).

Syndromic respiratory testing, while typically reserved for inpatients or those likely to be admitted at our institution, may also help reduce inpatient admissions when disposition is unclear. Given its faster and more consistent TAT, the RPP was more frequently ordered in the ED than the RVP. Fewer patients with positive RPPs were admitted, whereas the proportion of admitted patients with negative tests was equivalent in both the RVP and the RPP groups. Rapid confirmation of a viral etiology may increase provider comfort with discharge from the ED.

We noted an 8.4% drop in hospital admissions for RPP-positive patients in our EDs, accounting for approximately 31 avoided admissions over the 2-month study period. In this time, 1,043 RPPs were performed on adult patients with respiratory viral symptoms within 48 h of ED presentation, with a cost of approximately $156,450 (∼$150 per RPP). At an estimated national average of $7,282 per inpatient admission stay for community-acquired pneumonia (46), preventing just 22 unnecessary admissions would have accounted for the cost of performing the RPP in this clinical setting. This estimation does not account for potential cost savings associated with reduced antibiotic usage, including reduced pharmacy costs, the need for laboratory monitoring of antibiotic levels when indicated, or possible C. difficile infection. Rapid molecular detection of respiratory pathogens, while initially criticized for its expense, has been identified as a potential resource and cost saving intervention (18, 24, 40, 47–51). In addition, syndromic respiratory testing expedites patient cohorting, optimization of isolation rooms (17, 27, 52, 53), and increased ability to track epidemiologic trends (48).

Our study has a number of limitations. Due to its retrospective nature, confounding may be present. LOT was defined as discrete days in which antibiotics were received. Thus, differences in antibiotic duration at smaller intervals may have gone undetected. Only patients with positive tests results admitted to our hospitals were selected for primary analysis, not allowing clinical comparisons to those admitted with negative test results. We did not further evaluate chest imaging that was deemed abnormal, thus potentially overemphasizing the impact of minimal radiographic changes in subjects with abnormal imaging. However, this method was the most conservative for establishing normal imaging. We noted an increase in the detection of influenza A by the RPP, likely because fewer initial rapid influenza tests were performed in this population compared to the RVP. The rapid influenza PCR was available during both seasons. Asthma was more prevalent in the RPP-positive group, possibly reflecting an increasing use of the RPP for cohorting; however, the proportions of patients with asthma receiving antibiotics were not significantly different (50 and 51.8%). ED physicians were recommended to only order the RVP or RPP in patients who were likely to be admitted; thus, it is possible that the lower likelihood of admission in the RPP-positive group reflects ED physicians poorly predicting which patients were likely to be admitted. However, admission rates were similar among patients with negative test results. More patients in the RVP group were admitted to the ICU; however, accounting for a higher level of care did not change the conclusions of our primary outcomes. Finally, there was no difference observed in 30-day readmission, in-hospital mortality, or positive C. difficile test results, likely due to the low frequency of these events.

In conclusion, implementation of the RPP in the adult population provided results fast enough to be clinically actionable, reducing inpatient admissions, and, when combined with negative chest imaging, a positive test result was associated with reduced initiation of inappropriate antibiotics. In cases of diagnostic uncertainty for whom there is still the challenge of distinguishing respiratory viral infection from bacterial coinfection, the RPP should be coupled with input from diagnostic management teams and antibiotic stewardship programs.
